# Insights into the role of MSLN-positive circulating tumor cell as an auxiliary diagnostic biomarker in epithelial ovarian cancer

**DOI:** 10.3389/fonc.2025.1563095

**Published:** 2025-07-28

**Authors:** Hang Xu, Min Wang, Shuting Wu, Qinke Li, Jinlong Wang, Siying Zhang, Qiongming Liu, Mengting Wang, Ruifang Li, Zhiyuan Hu, Yi Liu, Zhu Yang

**Affiliations:** ^1^ Department of Gynecology and Obstetrics, the Second Affiliated Hospital of Chongqing Medical University, Chongqing, China; ^2^ Fujian Provincial Key Laboratory of Brain Aging and Neurodegenerative Diseases, School of Basic Medical Sciences, Fujian Medical University, Fuzhou, Fujian, China; ^3^ Nanopep Biotech Co., Beijing, China; ^4^ Department of Biochemistry, Shanxi Medical University, Taiyuan, Shanxi, China; ^5^ CAS Key Laboratory for Biomedical Effects of Nanomaterials and Nanosafety, CAS Key Laboratory of Standardization and Measurement for Nanotechnology, CAS Center for Excellence in Nanoscience, National Center for Nanoscience and Technology, Beijing, China

**Keywords:** MSLN, circulating tumor cells, epithelial ovarian cancer, diagnosis, EpCAM (CD326)

## Abstract

**Background:**

Epithelial ovarian cancer (EOC) currently lacks highly specific biomarkers for clinical screening. This study aimed to identify and validate novel auxiliary diagnostic markers for EOC.

**Methods:**

Through integrated analysis of transcriptome sequencing data and single-cell RNA sequencing from public databases, we identified mesothelin (MSLN) as an EOC-specific target. MSLN expression was subsequently validated in EOC cell lines and clinical specimens by flow cytometry, immunofluorescence, and immunohistochemistry. The capture efficacy of Pep@MNPs (Magnetic nanoparticles functionalised with EpCAM peptides) on EOC cells was verified by scanning electron microscopy, Prussian blue staining and cell spiked-blood capture experiments. In a prospective cohort of 35 patients with undiagnosed ovarian masses, we employed immunofluorescence staining to detect MSLN-positive circulating tumor cells (MSLN(+)CTCs) and assessed their diagnostic performance using receiver operating characteristic (ROC) analysis.

**Results:**

MSLN was highly expressed in EOC cell line and tissues but lowly expressed in normal ovarian surface epithelial tissues. EOC cells can be captured by Pep@MNPs with high sensitivity and specificity. ROC curves analysis showed that MSLN(+)CTCs differentiated between benign and malignant lesions of the ovary with a sensitivity of 66.67% and a specificity of 95% (p = 0.0014), which was more specific than cancer antigen 125 (CA125) (sensitivity: 71.43%; specificity: 94.47%; p < 0.0001) and human epididymis protein 4 (HE4) (sensitivity: 84.62%; specificity: 89.47%; p = 0.0002). When MSLN(+)CTCs were combined with CA125, the sensitivity was 92.86% and the specificity was 94.74%, p < 0.0001, which greatly improved the diagnostic sensitivity while preserving high specificity.

**Conclusions:**

MSLN(+)CTCs represent a highly specific auxiliary biomarker for differentiating benign and malignant ovarian lesions. The combination of MSLN(+)CTCs with CA125 provides an optimal balance between sensitivity and specificity, offering promising clinical utility for EOC diagnosis.

## 1 Introduction

Ovarian cancer (OC) represents a significant global health burden, ranking as the eighth most prevalent malignancy among women worldwide while maintaining the highest mortality rate among gynecological cancers (1). Recent epidemiological data from 2020 reported 313,959 new OC cases globally, with 207,252 attributable deaths, underscoring its substantial impact on women’s health (2). Epithelial ovarian cancer (EOC) is the most common type of OC, accounting for around 90% of cases (3). The disease is frequently diagnosed at advanced stages (International Federation of Gynecology and Obstetrics [FIGO] stages III-IV) due to both nonspecific early clinical manifestations and the current absence of reliable screening modalities (4). Even following cytoreductive surgery and post-operative chemotherapy (5), the prognosis remains poor, with most patients experiencing disease recurrence within 36 months of initial treatment and five-year survival rates stagnating at 20%-50% (3, 4).

The clinical differentiation between benign and malignant adnexal masses is critical for determining appropriate therapeutic management (6). Currently, cancer antigen 125 (CA125) serves as the primary serum biomarker for OC detection (7). However, its diagnostic utility is constrained by several limitations: approximately 50-60% of early-stage (FIGO stages I-II) OC cases do not exhibit elevated CA125 levels (7, 8), while false-positive elevations are frequently observed in various benign gynecological conditions (9–11) including endometriosis, adenomyosis, uterine fibroids, and pelvic inflammatory disease. Another commonly used clinical serologic biomarker for OC detection as well as differential diagnosis, Human epididymis protein 4 (HE4), a member of the whey acidic four disulfide bond core (WFDC) protein family, shows elevated expression across multiple malignancies (ovarian, breast, endometrial, and lung cancers, as well as mesotheliomas) (7). Although HE4 is useful in identifying endometriosis with elevated CA125, HE4 levels remain affected by adenomyosis, tobacco use, and oral contraceptive administration (12). In addition, HE4 is not expressed at high levels in clear cell carcinoma of the ovary (7). These diagnostic challenges underscore the urgent need for highly specific biomarkers to improve OC detection and differential diagnosis.

Circulating tumor cells (CTCs) represent malignant cells that detach from primary or metastatic tumor sites and enter the peripheral circulation (13, 14). CTCs analysis offers several clinical advantages, including minimal invasiveness, real-time monitoring capability, and the potential for serial assessment of treatment response. Numerous studies have established the diagnostic and prognostic value of CTCs in various malignancies, including lung, breast, gastric, pancreatic, prostate and colorectal cancers (15–24). The isolation of CTCs presents significant technical challenges due to their extreme rarity in peripheral blood, with an estimated frequency of only 1 CTC per 10⁶ leukocytes (25). Currently, isolating CTCs from the blood of OC patients relies on the physical characteristics of tumor cells, such as density, size, and deformability, as well as the biological characteristics of the tumor, such as the expression of tumor markers or surface markers (epithelial cell marker EpCAM is currently the most commonly used). The CTCs in the enriched sample are mainly detected by immunocytochemistry (ICC) (26, 27) and reverse transcription polymerase chain reaction (RT-PCR) (28, 29). Fluorescence *in situ* hybridization (FISH) has also been reported for the identification of CTCs in ovarian cancer with stem cell-like fusion genes (30).

Mesothelin (MSLN) is a glycosylphosphatidylinositol (GPI)-anchored cell surface glycoprotein that is encoded by the *MSLN* gene. MSLN shows significant overexpression in multiple malignancies, including ovarian cancer, mesothelioma, pancreatic adenocarcinoma, and non-small cell lung cancer, and MSLN is expressed at low levels in normal tissues (31, 32). Its high expression in ovarian cancer is thought to be associated with cell adhesion, invasion, drug resistance, tumor progression, and peritoneal metastasis (33–35). Previous studies have utilized multiantibody-modified magnetic nanoparticles targeting MSLN, EpCAM, and N-cadherin for the isolation and molecular analysis of circulating tumor cells in epithelial ovarian cancer (36). Building on this foundation, we established a novel immunofluorescence staining technique to detect MSLN(+)CTCs in EOC by ICC and used it to distinguish between benign and malignant ovarian masses.

## 2 Materials and methods

### 2.1 Patients and samples

Between June and October 2024, 40 female patients with adnexal masses from the First Affiliated Hospital of Chongqing Medical University were enrolled in the study. All patients signed an informed consent form, and the study was approved by the Institutional Ethics Committee of the First Affiliated Hospital of Chongqing Medical University (approval nos. 2024-197-01).

The inclusion criteria for patients included: (1) age ≥ 20 years; (2) patients with undiagnosed new adnexal masses; and (3) voluntary participation in the study.

The exclusion criteria included: (1) histological diagnosis of non-epithelial ovarian cancer; (2) coexistence of other primary malignant tumors; and (3) any anti-cancer treatment within 6 months prior to enrollment, such as: (a) Chemotherapy: Platinum-based regimens (e.g., carboplatin/paclitaxel) or dose-dense paclitaxel; (b) Targeted therapy: Poly ADP-ribose polymerase (PARP) inhibitors (e.g., olaparib) or anti-angiogenic agents (e.g., bevacizumab); (c) Immunotherapy: Immune checkpoint inhibitors (e.g., Programmed Cell Death Protein 1/Programmed Death-Ligand 1 (PD-1/PD-L1) inhibitors); (d) Radiotherapy: Palliative radiation; (e) Hormonal therapy: Aromatase inhibitors (e.g., letrozole).

2 mL of peripheral blood was collected from each patient before primary cytoreductive surgery in CTC-specific blood collection tubes (Nanopep Biotech, Beijing) for CTCs detection. CTC-specific blood collection tubes contain paraformaldehyde for fixing CTCs. The blood samples were transported and stored at 25°C and processed within 3 days.

Serum levels of CA125 and HE4 were derived from official laboratory test reports issued by the Department of Laboratory Medicine, the First Affiliated Hospital of Chongqing Medical University.

### 2.2 Target gene selection and validation

Our data were obtained from multiple publicly available databases. Transcriptome data were taken from Cancer Genome Atlas (TCGA), which contains 425 EOC samples, and the Genotypic Tissue Expression (GTEx) database, which includes 88 normal ovarian epithelial tissue samples. All expression data were downloaded from the University of California Santa Cruz (UCSC) Xena platform, normalized, batch corrected and retained as TPM (transcripts per million) values to ensure comparability. For single-cell analysis, we utilized the Tumor Immunotherapy Gene Expression Resource (TIGER) database, which provides detailed cell expression profiles for 2 paracancerous and 5 ovarian tumor samples. For differential gene expression analysis between tumor and normal samples (TCGA vs. GTEx), we applied the threshold |logFC| > 1 with a significance level of p < 0.05. In addition, in the TCGA cohort, we performed separate analyses of samples from distantly metastatic and non-metastatic patients using the same statistical threshold. To further refine our list of candidate genes, we improved stringency by applying a more stringent fold change criterion (|logFC| > 3, p < 0.05). From the single-cell analysis, we identified tumor cell-specific genes by comparing the expression profiles between tumor cells and non-tumor cells in the microenvironment. Gene intersections identified by these complementary approaches were further evaluated using the Gene Expression Profiling Interactive Analysis (GEPIA) online tool (http://gepia.cancer-pku.cn/index.html) to assess their expression distribution in 31 different tumor types. After screening the candidate genes, we identified MSLN as the study gene. After that, we also selected 285 ovarian tumor samples from the Gene Expression Omnibus (GEO) database to analyze the relationship between MSLN expression levels and ovarian cancer stages and grades. The validation of MSLN was mainly carried out from the aspects of cell lines and tissues.

### 2.3 Cell lines

The OVCAR3, CAOV3, SKOV3 and A2780 OC cell lines were purchased from Wuhan Pricella Biotechnology Co., Ltd. OVCAR3 was cultured in OVCAR3 Cell Complete Medium (CM-0178; Pricella, Wuhan), composed of RPMI-1640 supplemented with 20% fetal bovine serum (FBS), 10 μg/mL insulin, and 1% penicillin/streptomycin. SKOV3 was cultured in SKOV3 Cell Complete Medium (CM-0215; Pricella, Wuhan), composed of McCoy’s 5A supplemented with 10% FBS and 1% penicillin/streptomycin. CAOV3 and A2780 were grown in DMEM (Gibco, USA) supplemented with 10% FBS and 1% streptomycin in a 37°C incubator with 5% CO_2_. Peripheral blood mononuclear cells (PBMCs) were obtained from the peripheral blood of healthy volunteers and was extracted using Human Peripheral Blood Lymphocyte Isolation Solution (LTS1077; TBD), which was operated in strict accordance with the instructions.

### 2.4 Flow cytometry

The four OC cell lines and PBMCs were resuspended in phosphate buffered saline (PBS), fixed with 4% paraformaldehyde (4% PF) for 10 minutes at 25°C, centrifuged and washed with PBS, then blocked with 5% bovine serum albumin for 30 minutes. After that, the cells were incubated with FITC anti-human CD326 (EpCAM) Antibody (324203; 1:50; BioLegend) and Alexa Fluor^®^ 647 Anti-Mesothelin Antibody (ab252135; 1:500; Abcam) in the dark for 60 minutes, washed with PBS, and then detected using a flow cytometer (BD FACSCelesta™). Data were analyzed by Flowjo software (Tree Star).

### 2.5 Immunofluorescence staining

The four OC cell lines and PBMCs were fixed with 4% PF for 10 minutes. After that, they were blocked with 5% bovine serum albumin for 30 minutes. After blocking, the cells were incubated with FITC anti-human CD326 (EpCAM) Antibody (324203; 1:50; BioLegend) and Alexa Fluor^®^ 647 Anti-Mesothelin Antibody (ab252135; 1:500; Abcam) for 60 minutes in the dark, washed with PBS, and the nuclei were stained with 4′,6-diamidino-2-phenylindole (DAPI; P0131; Beyotime). The immunofluorescence images were observed and collected using a fluorescence microscope (HDS-FLS-IX73-STD-1C) and processed and quantitatively analyzed by ImageJ software.

### 2.6 Immunohistochemistry

In 2024, 10 paraffin-embedded EOC and 5 normal ovarian surface epithelial tissues samples were collected from the Department of Pathology of the First Affiliated Hospital of Chongqing Medical University. The tissue sections were torrefied for 2 hours, deparaffinized using xylene and rehydrated using anhydrous ethanol. Antigen repair was performed by heating the sections in citrate solution and then cooling to room temperature. The sections were then treated with 3% hydrogen peroxide solution for 10 minutes at room temperature to block peroxidase activity. After rinsing, the sections were stained with MSLN (66404-1-lg; Proteintech; Wuhan) and EpCAM antibodies (66316-1-lg; Proteintech; Wuhan) separately, 3,3’-diaminobenzidine was used to visualization, and the sections were counterstained with hematoxylin, sealed, and the images were captured using a high-resolution optical system. IHC scores were based on the percentage of positive cells (0, < 5%; 1, 5%-25%; 2, 26%-50%; 3, 51%-75%; 4, > 75%) as well as the intensity of staining (0, negative; 1, weakly positive; 2, moderately positive; 3, strongly positive). Total score = percentage of positive cells score × intensity of staining score.

### 2.7 Scanning electron microscopy characterization of isolated cells

Magnetic nanoparticles functionalised with EpCAM peptides (Pep@MNPs) and bare magnetic nanoparticles (MNPs) (Nanopep Biotech, Beijing) were incubated with ovarian cancer cell line OVCAR3 and human T-cell leukemia cell line Jurkat (Nanopep Biotech, Beijing, Gift) at 25°C for 60 minutes. The cells were allowed to settle on the silicon wafer for 60 minutes, fixed with 4% paraformaldehyde for 10 minutes, dehydrated with a gradient of ethanol (10%, 30%, 50%, 70%, 90%, 100%, 100%), and finally dried in an incubator at 37°C. The morphology of the cells captured by Pep@MNPs was characterized using a thermal field-emission environmental scanning electron microscope(S-4800).

### 2.8 Prussian blue staining

OVCAR3 cells were incubated with Pep@MNPs and MNPs at 25°C for 60 minutes, and then allowed to settle on the glass slides for 30 minutes. Afterward, they were stained using the Prussian Blue Iron Staining Kit (G1426; Solarbio), strictly following the steps in the instructions. After sealing the slides, they were observed under a microscope and photographed.

### 2.9 Sensitivity investigation of the Pep@MNPs toward OVCAR3

We diluted 2 mL of whole blood from healthy volunteers in a 15 mL centrifuge tube with PBS to 10 mL, then incubated OVCAR3 with Hoechst33342 staining solution (C0030; Solarbio) at 37°C for 30 minutes in the dark. After that, it was washed three times with PBS, counted using a hemocytometer and then we spiked OVCAR3 into diluted whole blood according to a quantitative gradient (100, 500, 1000, 2000, 7000), added 10 μL Pep@MNPs, mixed well in a shaker at 25°C for 60 minutes, and then used a magnetic rack (Nanopep Biotech, Beijing) for 30 minutes. After that, the enrichment product was washed twice with PBS, and the enrichment product was allowed to settle on a glass slide for 60 minutes. The sample was scanned and counted using the TUMORFISHER^®^ CTC detection platform (Nanopep Biotech, Beijing).

### 2.10 Specificity capture test

We selected the EpCAM-high-expressing cell line OVCAR3, the EpCAM-low-expressing cell lines A2780 and SKOV3, and the human T-cell leukemic line Jurkat for the capture experiment. The cells were first incubated with Hoechst 33342 staining solution at 37°C for 30 minutes in the dark, then washed three times with PBS. After that, the cells (Each cell line was spiked into 7000) were spiked into 10 mL of whole blood from a healthy volunteer that had been diluted in advance, and 10 μL of Pep@MNPs were added. The sample was mixed in a shaker for 60 minutes, then enriched on a magnetic rack for 30 minutes, after which the enrichment product was washed twice with PBS, and finally the enrichment product was allowed to settle on a glass slide for 60 minutes. The sample was scanned and counted using the TUMORFISHER^®^ CTC detection platform.

### 2.11 Exploration of the optimal dilution ratio of MSLN antibody

We mixed 5000 OVCAR3 with the whole blood of healthy volunteers (100 μL whole blood, 400 μL PBS for dilution, total 500 μL), fixed it with 500 μL 4% paraformaldehyde for 10 minutes, centrifuged to remove the supernatant, washed it three times with PBS, blocked it with 5% bovine serum albumin for 30 minutes, centrifuged to remove the supernatant, incubated the cells with different dilutions (1:100, 200, 300, 400, 500) of Anti-Mesothelin Antibody at 25°C for 60 minutes in the dark, after which the cells were washed three times with PBS, centrifuged, resuspended in PBS, and then deposited on a glass slide for 30 minutes. After aspirating the supernatant, add DAPI to stain the nuclei, seal the slides, and then scan the samples using the TUMORFISHER^®^ CTC detection platform to count the fluorescence intensity of MSLN expression.

### 2.12 OVCAR3 mixed with whole blood for staining

We mixed 5000 OVCAR3 with the whole blood of healthy volunteers (100 μL whole blood, 400 μL PBS for dilution, total 500 μL), fixed it with 500 μL 4% paraformaldehyde for 10 minutes, centrifuged to remove the supernatant, washed three times with PBS, permeabilized with 100 µL anhydrous ethanol (immediately aspirated and air-dried), then blocked with 5% bovine serum albumin for 30 minutes, centrifuged to remove the supernatant, and then incubated the cells with cytokeratin antibody mixture (CK8, 18, 19, C-003-A, C-004-A, Nanopep Biotech, Beijing), CD45 antibody (C-001-A, Nanopep Biotech, Beijing), Alexa Fluor^®^ 647 Anti-Mesothelin Antibody (ab252135; Abcam) in the dark at 25°C for 60 minutes. After incubation, the cells were washed three times with PBS, centrifuged, resuspended in PBS, and then deposited on a glass slide for 30 minutes. The supernatant was aspirated, and the cells were stained with DAPI to stain the nuclei. OVCAR3 staining in PBS remained consistent with the above steps exactly except for the absence of blood. Finally, the slides were sealed and the samples were scanned using the TUMORFISHER^®^ CTC detection platform. The mean fluorescence intensity (MFI) of the cells in the immunofluorescence images was quantified using ImageJ software.

### 2.13 Enrichment and identification of CTCs

10 µL of pre-vortexed Pep@MNPs (C-006-A, Nanopep Biotech, Beijing) were added to 2.0 mL of peripheral blood samples, diluted to 10 mL with PBS, and gently shaken for 1 h at 25°C. Captured CTCs were subsequently separated by a magnetic field for 30 min, washed twice with PBS, and the enriched products were fixed with 4% PF for 30 min, permeabilized with 100 µL anhydrous ethanol (immediately aspirated and air-dried), then blocked with 5% bovine serum albumin for 30 min, and then stained with polyclonal antibodies, including DAPI for nuclear staining, Alexa Fluor 488-cytokeratin antibody mixture (CK8, 18, 19, C-003-A, C-004-A, Nanopep Biotech, Beijing) for positive selection and Alexa Fluor 594-CD45 antibody (C-001-A, Nanopep Biotech, Beijing) for negative selection (leukocytes) and Alexa Fluor^®^ 647 Anti-Mesothelin Antibody (ab252135; Abcam) for MSLN expression characterization. Cytokeratin and CD45 antibodies were diluted strictly according to instructions and the MSLN staining dilution ratio was 1:300. CTCs were identified under an Imager Z2 fluorescence microscope (Carl Zeiss, Jena, Germany) based on molecular characterization (CKmix+/DAPI+/CD45-/MSLN+), (CKmix+/DAPI+/CD45-). Cytokeratin (CK, an epithelial cell marker) and CD45 (leukocyte common antigen) were identified markers for CTCs of epithelial tumor origin, which were molecularly characterized as CK+/DAPI+/CD45- and leukocytes as CK-/DAPI+/CD45+ under fluorescence microscopy. The TUMORFISHER CTC detection platform is a platform that uses EpCAM peptide-functionalized magnetic nanoparticles to specifically recognize EpCAM on the surface of CTCs, then separates them under magnetic force, and finally identifies them using immunofluorescence staining.

The CTC detection process is divided into two steps, the first step is the automatic scanning fluorescence microscope scanning. The TUMORFISHER^®^ CTC detection platform performs an initial screening of whole target cells. The second step is manual selection, and the criteria for manual selection are as follows: MSLN-positive CTC has nuclei greater than or equal to 47 μm², the total fluorescence intensity of MSLN and CKmix of the target cell must be greater than or equal to the average of the total fluorescence intensity of MSLN and CKmix of the cells on the entire slide, and the total fluorescence intensity of CD45 of the target cell must be lower than the average of the total fluorescence intensity of CD45 of the cells on the entire slide. The cells in the fluorescence image are relatively clear and morphologically intact, with no background nonspecific staining, no halos, scattered distribution of the selected cells, and no black frame obscuring the field of view. Manual selection results are reviewed by a second person.

### 2.14 Statistical analysis

We performed normality tests on all data prior to statistical analysis. The basic characteristics of the enrolled patients were presented using descriptive statistics, and the Mann-Whitney *U* test was used to compare the difference in counts between MSLN-positive CTCs and CTCs in patients with EOC and benign ovarian disease. Diagnostic efficacy and cutoff values for MSLN-positive CTCs and CTCs were assessed using receiver operating characteristic (ROC) curves and the Youden index. All statistical analyses were performed using GraphPad Prism software (version 9, GraphPad Software, USA), p < 0.05 was considered statistically significant.

## 3 Results

### 3.1 MSLN is a relatively specific gene for EOC

**Figure 1:**
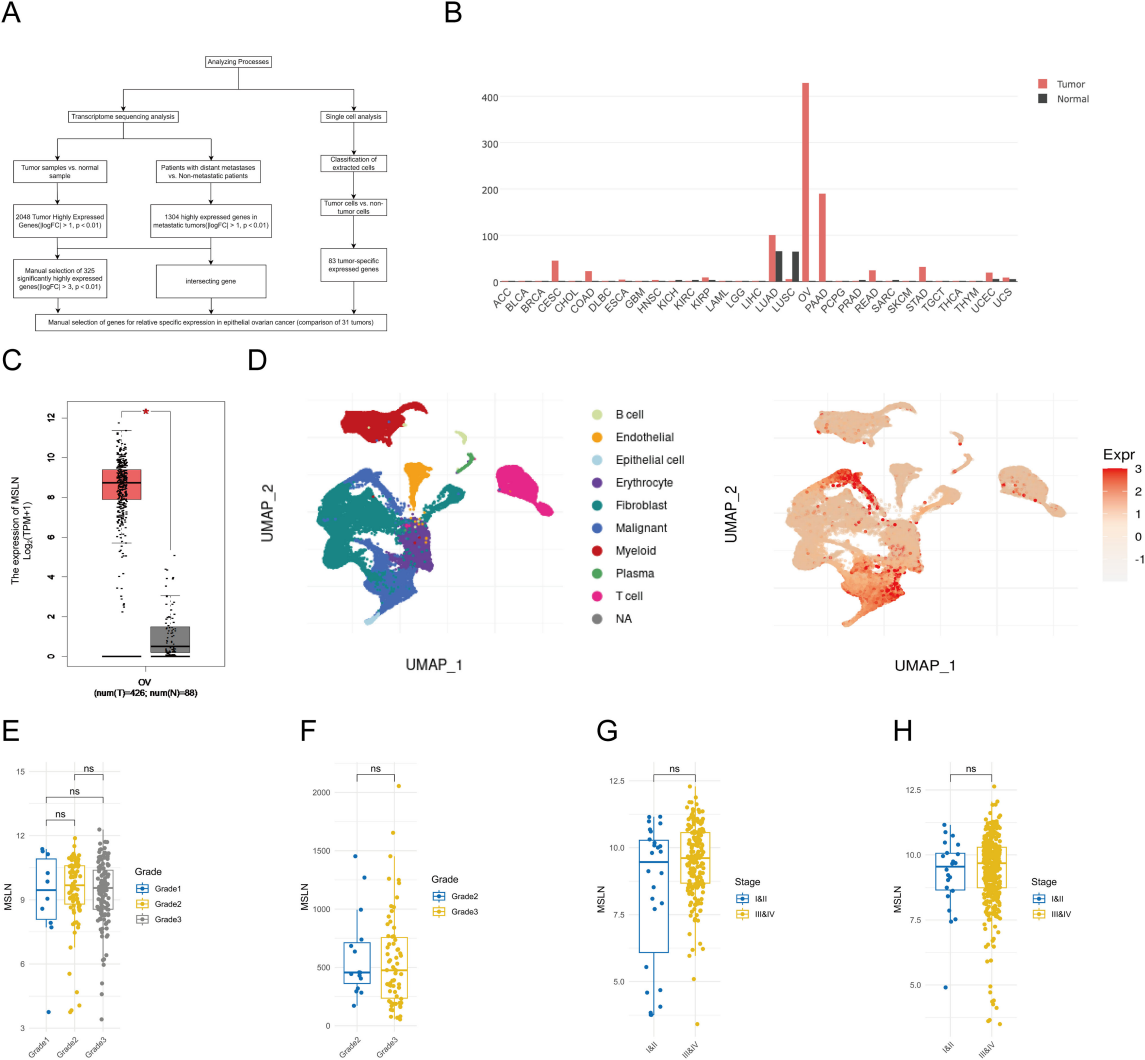
Selection and validation of MSLN as a biomarker specific for epithelial ovarian cancer (EOC). **(A)** Flowchart illustrating the systematic approach to identify EOC-specific genes by transcriptome sequencing analysis and single-cell analysis. **(B)** Expression profiles of MSLN in multiple cancer types (red bars, TCGA data) and their corresponding normal tissues (black bars, GTEx data). X-axis shows tissue abbreviations from TCGA, where “OV” stands for ovarian cancer. Other abbreviations represent different cancer types. Y-axis shows MSLN expression in TPM. **(C)** Box plot comparing MSLN expression levels between EOC tissues (left box, TCGA dataset, n=425) and normal ovarian tissues (right box, GTEx dataset, n=88), y-axis represents log2 transformed MSLN expression values. **(D)** UMAP visualization of single-cell data showing different cell types in ovarian tissues (left plot), where cell types are represented by different colors according to the legend, and MSLN expression distribution (right plot), where expression levels are represented by color intensities (red=high, blue=low) according to the expression scale. **(E)** Box line plot showing MSLN expression in different grades of ovarian cancer in the GEO database, where grade 1 is blue, grade 2 is yellow and grade 3 is gray. **(F)** Box line plot of MSLN expression for different grades of EOC in the TCGA database, where grade 2 is blue and grade 3 is yellow. **(G)** box line plot of MSLN expression at different clinical stages in the GEO database, comparing early (I+II, blue) with late (III+IV, yellow). **(H)** Box line plot of MSLN expression at different clinical stages in the TCGA database comparing early (I+II, blue) with late (III+IV, yellow). *p < 0.05, ns, not significant, p > 0.05.

To identify genes specifically expressed in EOC, we implemented a multistep selection approach combining bulk RNA sequencing and single-cell analysis (**Figure 1A**). First, we performed differential expression analysis on 425 EOC samples from TCGA and 88 normal ovarian tissue samples from GTEx (**Supplementary Table S1**), and identified 2048 significantly up-regulated genes in tumor samples (|logFC| > 1, p < 0.01). Also, a comparison of patients with distant metastases and those without metastases revealed 1304 significantly up-regulated genes in metastatic tumors (|logFC| > 1, p < 0.01). Given the large number of differentially expressed genes identified, we increased the stringency threshold to |logFC| > 3 (p < 0.01), which resulted in 325 genes being highly up-regulated in the EOC compared to normal tissue. Meanwhile, through single-cell analysis of the TIGER database (**Supplementary Table S2**), we identified 83 genes with specific expression in tumor cells but negligible expression in non-tumor cells within the tumor microenvironment. We then intersected these gene sets and performed manual selection based on the intersection results. After evaluating the expression patterns of the candidate genes in 31 tumor types using the GEPIA database (**Supplementary Figure S1**), we found that MSLN is expressed much higher in OV than in other types of tumors and MSLN expression was negligible in most normal tissues, with the exception of low-level expression observed in normal tissues corresponding to lung adenocarcinoma (LUAD, 65.68 TPM) and lung squamous cell carcinoma (LUSC, 64.68 TPM). [**Figures 1B, C**, the values of MSLN expression levels in different malignant tumor tissues in **Figure 1B** were (from high to low): OV 428.73 TPM, PAAD 189.75 TPM, LUAD 100.3 TPM, CESC 45.05 TPM, STAD 31.66 TPM, READ 24.36 TPM, COAD 22.50 TPM, UCEC 19.21 TPM, KIRP 8.96 TPM, UCS 8.72 TPM, ESCA 4.05 TPM, LUSC 5.23 TPM, MSLN expression was negligible in other cancer types]. Single-cell analysis revealed that MSLN was predominantly expressed in tumor cells, with minimal expression in non-tumor cells (e.g., stromal or immune cells) within the tumor microenvironment (**Figure 1D**). In addition to this, we found that MSLN expression levels were not associated with ovarian cancer stages (FIGO stages) and grades using sample analyses from the GEO database and TCGA database (**Figures 1E–H**). The above results indicate that MSLN is a relatively specific gene for EOC.

### 3.2 MSLN is highly expressed in OVCAR3 cells and EOC tissues

**Figure 2:**
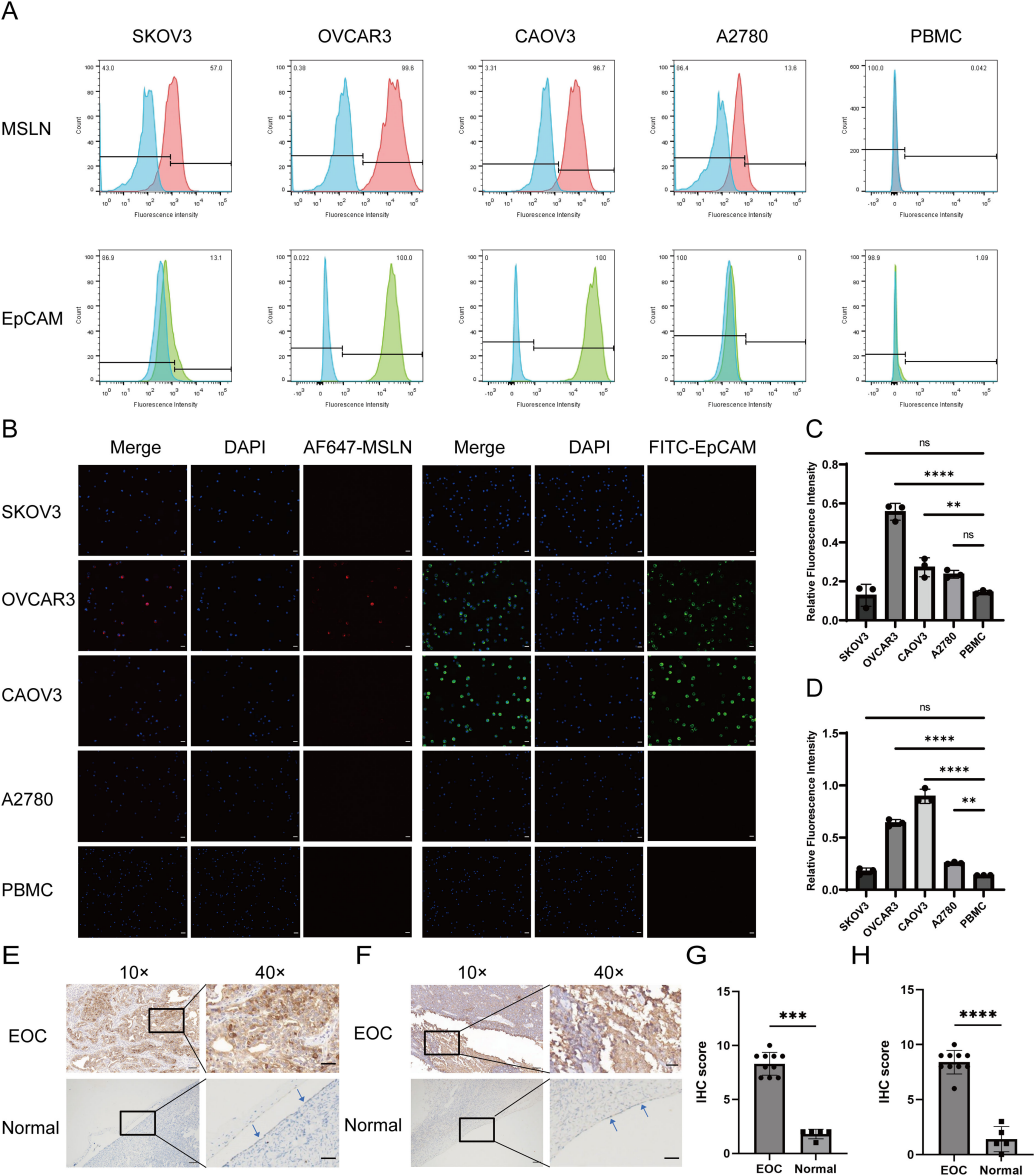
Validation of MSLN and EpCAM expression levels in OC cells and tissues. **(A)** The expression levels of MSLN and EpCAM on the surface of SKOV3, OVCAR3, CAOV3, A2780 and PBMC cells were verified by flow cytometry (n=3), where the red peak is the expression level of MSLN, the green peak is the expression level of EpCAM, and the blue peak is the unstained negative control. X-axis represents the fluorescence intensity and y-axis represents the ratio of the number of cells compared to the maximum count value. The percentage of negative cells is shown in the upper left corner of the graph, and the percentage of positive cells is shown in the upper right corner of the graph. **(B)** Immunofluorescence images show the expression levels of MSLN and EpCAM on the surface of SKOV3, OVCAR3, CAOV3, A2780, and PBMC cells (scale bar, 50μm). **(C)** Quantitative statistical plots of relative fluorescence intensity of MSLN expression levels on the surface of SKOV3, OVCAR3, CAOV3, A2780 and PBMC cells (n=3), x-axis is cell name and y-axis is relative fluorescence intensity. **(D)** Quantitative statistical plots of the relative fluorescence intensity of EpCAM expression levels on the cell surface of SKOV3, OVCAR3, CAOV3, A2780 and PBMC cells (n=3), x-axis is cell name and y-axis is relative fluorescence intensity. **(E)** Immunohistochemistry was performed to verify the expression levels of MSLN in epithelial ovarian cancer and normal ovarian surface epithelial tissue (indicated by the arrows, 10×, scale bar, 100μm; 40×, scale bar, 20μm). **(F)** Immunohistochemical verification of EpCAM expression levels in epithelial ovarian cancer and normal ovarian surface epithelial tissue (indicated by the arrows, 10×, scale bar, 100μm; 40×, scale bar, 20μm). **(G)** Quantitative statistical plots of MSLN expression levels in tissues (EOC, n=10; Normal, n=5). X-axis is the group and y-axis is the immunohistochemical score. **(H)** Quantitative statistical plots of EpCAM expression levels in tissues (EOC, n=10; Normal, n=5). X-axis is the group and y-axis is the immunohistochemical score. **p < 0.01, ***p < 0.001, ****p < 0.0001, ns, p > 0.05.

We selected four OC cell lines (OVCAR3, CAOV3, SKOV3, and A2780) along with PBMCs and evaluated the expression of MSLN and EpCAM by flow cytometry (**Figure 2A**). MSLN was detectable in all four OC cell lines, with particularly high expression observed in OVCAR3 cells. EpCAM expression levels varied among the cell lines, showing high expression in OVCAR3 and CAOV3 but low expression in SKOV3 and A2780. Notably, PBMCs exhibited negligible expression of both markers. These findings were further confirmed by immunofluorescence analysis (**Figures 2B–D**). Additionally, we performed immunohistochemical analysis on clinical specimens, including 10 epithelial ovarian cancer tissue samples and 5 normal ovarian surface epithelial tissue samples, to examine MSLN and EpCAM expression patterns in tissue contexts (**Figures 2E–H**). MSLN and EpCAM were found to have increased expression in EOC tissues and very low expression in normal ovarian surface epithelial tissues.

### 3.3 Pep@MNPs bind to OC cells and have a high capture rate

**Figure 3:**
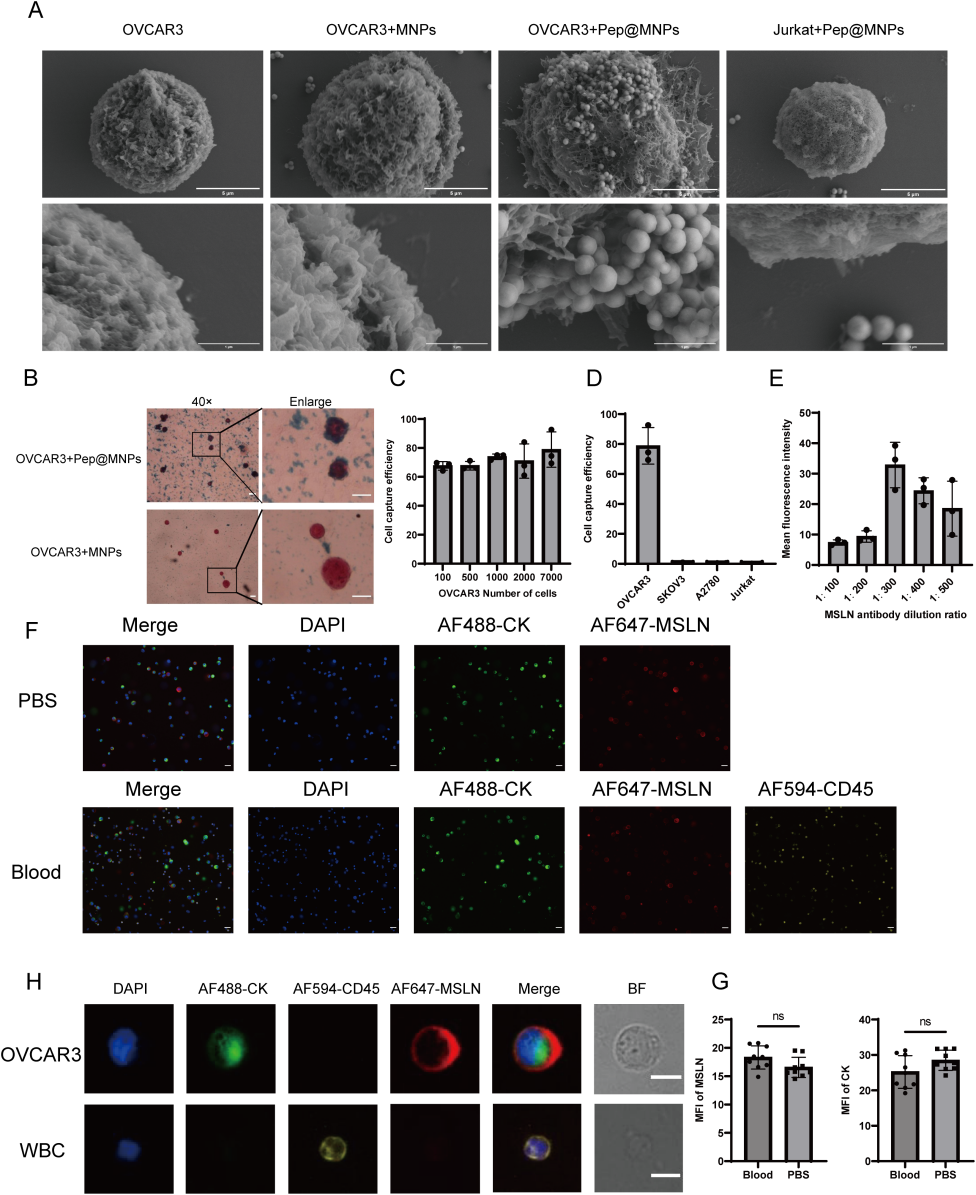
Validation of Pep@MNPs for OC cells capture efficiency and exploration of optimal dilution ratio of MSLN antibody. **(A)** Scanning electron microscopy images verifying the binding of Pep@MNPs to OC cells (n=3, scale bars, 5 μm and 1 μm). **(B)** Prussian blue-stained images verified the binding of Pep@MNPs to ovarian cancer cells (n=3, 40×, scale bar, 20 μm and enlarge, scale bar, 10 μm). **(C)** Capture efficiency of Pep@MNPs when OVCAR3 cells were spiked into blood in a number gradient of 100, 500, 1000, 2000, and 7000 (n=3). The x-axis is the number of spiked cells and the y-axis is the capture efficiency. **(D)** Capture efficiency of Pep@MNPs for ovarian cancer cells (OVCAR3, SKOV3, A2780) as well as leukocytes (Jurkat) spiked into blood (n=3). The x-axis is the cell name and the y-axis is the capture efficiency. **(E)** Mean fluorescence intensity of ovarian cancer cells when stained with different dilutions (1:100, 200, 300, 400, 500) of MSLN antibody (n=3). The x-axis is the antibody dilution ratio and the y-axis is the mean fluorescence intensity. **(F)** Immunofluorescence images of OVCAR3 staining in PBS environment and blood environment, respectively (n=3, scale bar, 50μm). **(G)** Statistical plots of the mean fluorescence intensity of OVCAR3 in the blood and PBS environments for CK and MSLN (Cells in 9 fields of view). The x-axis represents different staining environments and the y-axis represents the mean fluorescence intensity of gene on the cell surface in the field of view. The black dot represents the mean fluorescence intensity of the cells in one field of view. **(H)** Immunofluorescence images of individual OVCAR3 and WBC undergoing mixed staining in the blood environment. BF stands for Bright Field (n=3, scale bar, 10 μm). “ns”, p>0.05.

To evaluate the binding specificity of magnetic nanoparticles to OC cells, we selected the EpCAM-high-expressing OVCAR3 cell line and the human T-cell leukemia cell line Jurkat as a negative control. SEM revealed that Pep@MNPs selectively bound to OVCAR3 cells but not to Jurkat cells, whereas MNPs showed no binding to OVCAR3 cells (**Figure 3A**). This observation was further confirmed by Prussian blue staining, which demonstrated binding of Pep@MNPs to OVCAR3 but not of MNPs (**Figure 3B**). These results indicate that Pep@MNPs specifically target EpCAM-expressing OC cells without adhering to leukocytes, and that MNPs exhibit no nonspecific binding.

Next, we assessed the capture sensitivity and specificity of Pep@MNPs for OC cells. First, to determine sensitivity, we spiked Hoechst33342-prestained OVCAR3 cells into healthy donor peripheral blood at defined quantitative gradient (100, 500, 1000, 2000, 7000) and performed magnetic capture (**Figure 3C**). The capture efficiencies were 67.58%, 67.69%, 73.89%, 70.92%, and 78.81%, respectively, demonstrating high sensitivity across a broad dynamic range. To evaluate specificity, we compared the capture efficiency of Pep@MNPs for EpCAM-high (OVCAR3), EpCAM-low (SKOV3, A2780), and Jurkat cells spiked into peripheral blood (**Figure 3D**). The capture rates were 78.81% for OVCAR3, but only 0.51% for SKOV3, 0.36% for A2780, and 0.10% for Jurkat. These findings confirm that Pep@MNPs exhibit high specificity for EpCAM-expressing OC cells while minimally capturing leukocytes or EpCAM-low OC cells.

To determine the optimal staining conditions for MSLN detection, we performed a systematic evaluation of antibody dilution ratios using OVCAR3 cells spiked into blood. Anti-mesothelin antibody dilutions ratios ranging from 1:100 to 1:500 were tested (**Figure 3E**). Maximum MFI was achieved at a 1:300 dilution, while lower antibody dilution ratios (1:100-1:200) resulted in diminished fluorescence signals. This observed decrease in signal intensity at lower antibody dilution ratios may be attributed to three potential mechanisms: (1) antibody saturation leading to reduced binding efficiency and fluorescence quenching, (2) increased non-specific binding and elevated background signal, and (3) antigen-antibody complex formation favoring small soluble complexes over large precipitates at excessive antibody concentrations. Comparative analysis of staining efficiency in blood versus PBS environments demonstrated that OVCAR3 cells maintained robust MSLN and CK staining in blood, with no significant background interference (**Figure 3F**). Quantitative assessment revealed comparable MFI values for both MSLN and CK between blood and PBS conditions (**Figure 3G**). OVCAR3 showed CKmix+/DAPI+/CD45-/MSLN+ and leukocytes showed CKmix-/DAPI+/CD45+ under fluorescence microscopy (**Figure 3H**).

### 3.4 Patient enrollment

This prospective study initially screened 40 patients presenting with suspected ovarian cancer or undiagnosed ovarian masses (**Supplementary Figure S2**). Following application of stringent inclusion/exclusion criteria, we enrolled 35 patients for final analysis: 15 with EOC and 20 with benign ovarian lesions. Demographic analysis revealed no significant age difference between EOC patients (median: 56 years; range: 37-67) and benign cases (median: 46.5 years; range: 27-89) using Welch’s corrected t-test (**Supplementary Figure S3**). The clinical characteristics of the EOC cohort are detailed in **Supplementary Table S3**: within the 15 patients with EOC, 12 patients (80.0%) presented with symptoms at the time of diagnosis, including abdominal pain, abdominal distension, loss of appetite and vaginal bleeding, and 14 (93.3%) patients had CA125 ≥35U/ml, 6 (40.0%) patients had early stage (FIGO stages I and II) epithelial ovarian cancer, 4 (26.7%) patients showed lymph node involvement and 7 (46.7%) patients presented with peritoneal metastases. The histologic diagnosis of the patients was serous carcinoma in the highest number of patients (60.0%), followed by clear cell carcinoma (33.3%), and carcinosarcoma (6.7%).

### 3.5 Characterization and counting of CTCs as well as MSLN(+)CTCs

**Figure 4:**
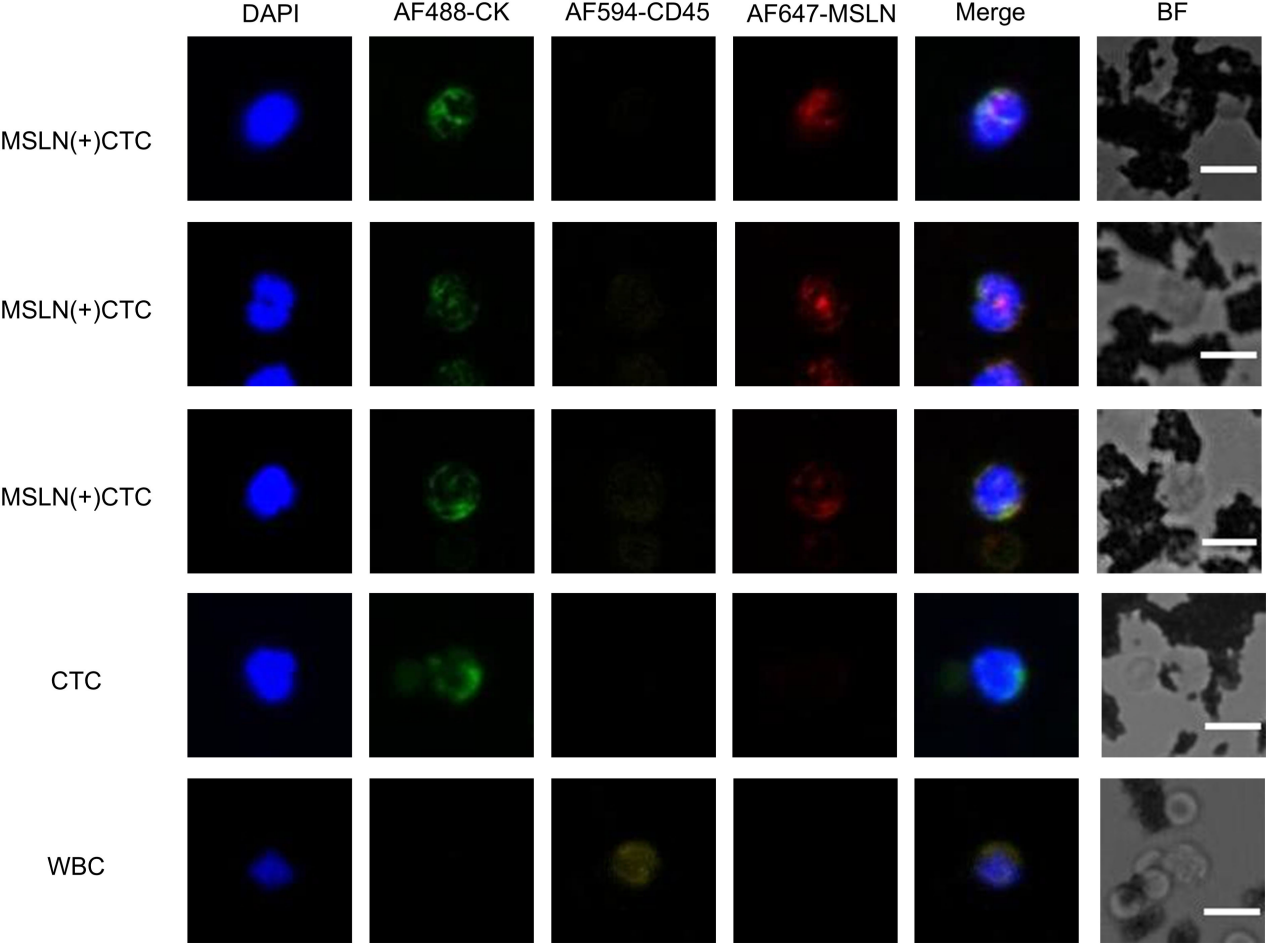
Immunofluorescence images of MSLN(+)CTC (MSLN-positive CTC), CTC, and WBC captured in clinical samples. Characterization: MSLN-positive CTC (CKmix+/DAPI+/CD45-/MSLN+), CTC (CKmix+/DAPI+/CD45-), WBC (CKmix-/DAPI+/CD45+). BF stands for Bright Field.

MSLN(+)CTCs as well as CTCs captured in patient samples were characterized and counted using the TUMORFISHER^®^ CTC detection platform. The molecular characterization of MSLN(+)CTCs under fluorescence microscopy was CKmix+/DAPI+/CD45-/MSLN+, that of CTCs was CKmix+/DAPI+/CD45-, and that of leukocytes was CKmix-/DAPI+/CD45+ (**Figure 4**). The counting results and CA125, HE4 levels are shown in **Table 1**, with a mean number of MSLN(+)CTCs of 2.7 (median: 1.0; range: 0.0-9.0; standard deviation: 2.9; 95% CI: 1.2-4.2) in patients with EOC and 0.1 (median: 0.0; range: 0.0-1.0; standard deviation: 0.2; 95% CI:0.0-0.1) in patients with benign ovarian lesions. Analysis using the Mann Whitney test revealed a significant difference in the number of MSLN(+)CTCs between the two groups (**Supplementary Figure S4A**). Meanwhile, the mean number of CTCs was 5.0 (median: 3.0; range: 0.0-13.0; standard deviation: 4.6; 95% CI: 2.7-7.3) in patients with EOC and 0.4 (median: 0.0; range: 0.0-2.0; standard deviation: 0.7; 95% CI: 0.0-0.7) in patients with benign ovarian lesions. A significant difference in the number of CTCs between the two groups of patients was found by Mann Whitney test analysis (**Supplementary Figure S4B**). Similarly, we found significant differences in CA125 and HE4 levels between the two groups by Mann Whitney test analysis (**Supplementary Figure S4C–D**). The detection rate of MSLN(+)CTCs in patients with EOC was 66.7%, and the detection rate of CTCs was 86.7%.

**Table 1:** MSLN(+)CTC, CTC counts and CA125, HE4 levels among different groups.

Variable	EOC (n=15)	Benign ovarian lesions (n=20)
Age median, years (range)	56(37-67)	46.5(26-89)
MSLN(+)CTC counts, cells		
Median	1.0	0.0
Mean	2.7	0.1
Standard deviation	2.9	0.2
Range (min-max)	0.0-9.0	0.0-1.0
95% CI	1.2-4.2	0.0-0.1
CTC counts, cells		
Median	3.0	0.0
Mean	5.0	0.4
Standard deviation	4.6	0.7
Range (min-max)	0.0-13.0	0.0-2.0
95% CI	2.7-7.3	0.0-0.7
CA125 levels, U/mL		
Median	792.4	20.0
Mean	1357.8	44.0
Standard deviation	1538.2	49.4
Range (min-max)	24.8-4217.5	6.2-156.9
95% CI	552.0-2163.6	21.8-66.2
HE4 levels, pmol/L		
Median	258.0	38.0
Mean	292.4	40.7
Standard deviation	251.9	15.4
Range (min-max)	27.0-681.0	25.0-85.0
95% CI	155.5-429.3	33.8-47.6

### 3.6 MSLN(+)CTCs and CTCs used for differentiating benign and malignant ovarian lesions

**Figure 5:**
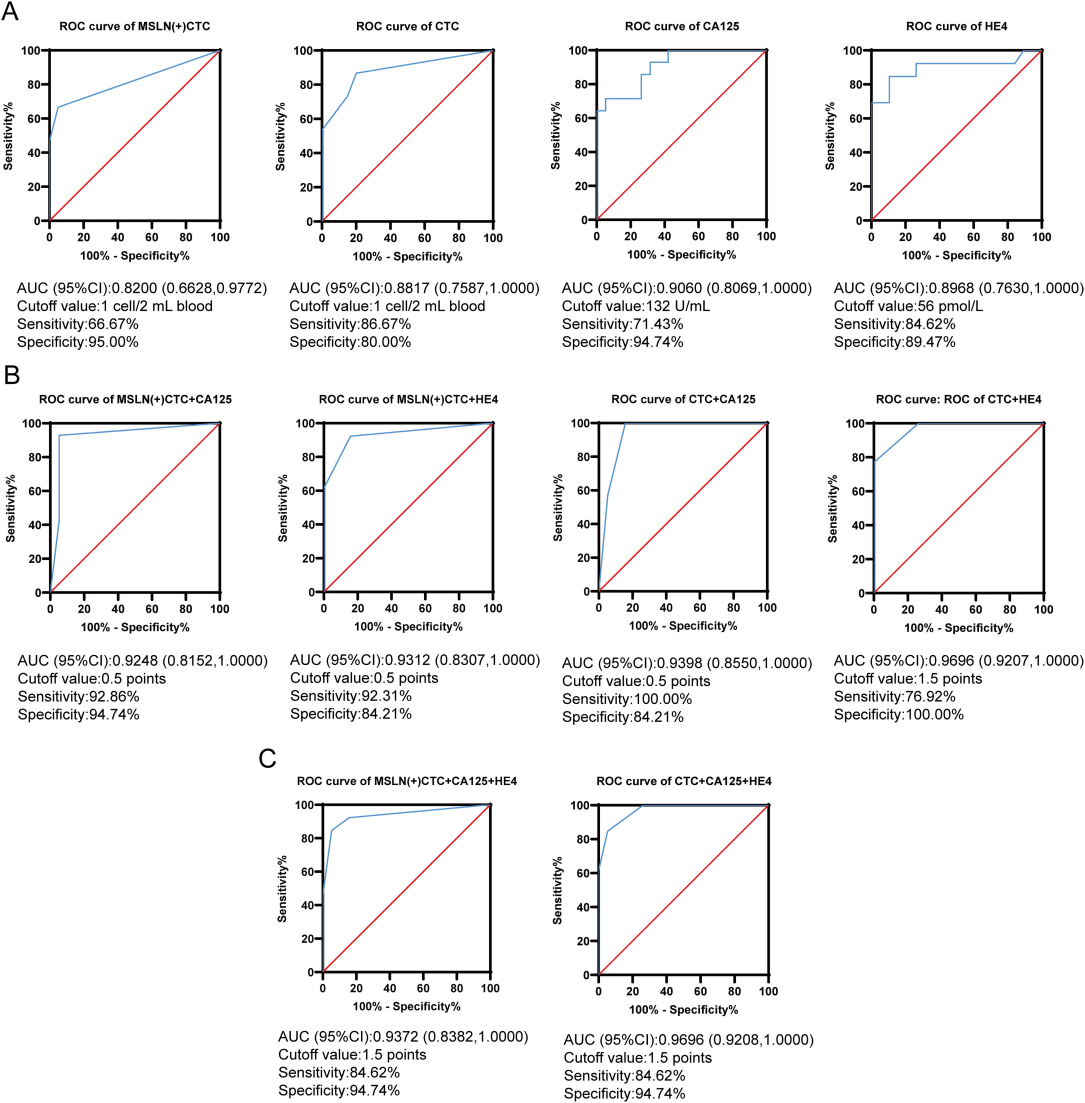
MSLN(+)CTC, CTC, CA125 and HE4 were used for differentiating benign and malignant ovarian lesions. **(A)** ROC curves for MSLN(+)CTC, CTC, CA125 and HE4 were used for differentiating benign and malignant ovarian lesions. The x-axis is the false positive rate, the y-axis is the true positive rate, the red line is the null curve, and the blue line is the ROC curve. **(B)** Dual indicators combined ROC curves used for differentiating benign and malignant ovarian lesions. The x-axis is the false positive rate, the y-axis is the true positive rate, the red line is the null curve, and the blue line is the ROC curve. **(C)** Triple indicators combined ROC curves used for differentiating benign and malignant ovarian lesions. The x-axis is the false positive rate, the y-axis is the true positive rate, the red line is the null curve, and the blue line is the ROC curve.

We assessed the efficacy of MSLN(+)CTCs, CTCs, CA125, and HE4 to differentiate benign and malignant ovarian lesions using the ROC curve and the Youden index, respectively (**Figure 5A**). The optimal cutoff value for MSLN(+)CTCs determined using the maximum Youden index was 1 cell/2 mL of blood, with an area under the ROC curve (AUC) of 0.820, p = 0.0014, and a sensitivity of 66.67% and specificity was 95%. The optimal cutoff value for CTCs was 1 cell/2 mL of blood, AUC was 0.882, p = 0.0001, sensitivity was 86.67%, and specificity was 80%. The optimal cutoff for CA125 was 132 U/mL, AUC 0.906, p < 0.0001, sensitivity 71.43% and specificity 94.74%. The optimal cutoff for HE4 was 56 pmol/L with an AUC of 0.897, p = 0.0002, sensitivity 84.62% and specificity 89.47%. Comparative analysis revealed that MSLN(+)CTCs demonstrated superior specificity (95%) compared to both HE4 (89.47%) and CTCs (80%), with marginally higher specificity than CA125 (94.74%). CTCs exhibited the highest sensitivity (86.67%) among all biomarkers. However, the diagnostic performance of individual indicators remained suboptimal. While MSLN(+)CTCs showed excellent specificity, this came at the expense of reduced sensitivity.

To improve the accuracy of diagnosis, we used MSLN(+)CTCs and CTCs as auxiliary diagnostic indicators in combination with CA125 and HE4 for differentiating benign and malignant ovarian lesions. We established a combined scoring diagnostic model. We first combined MSLN(+)CTCs and CTCs with CA125 and HE4, respectively. The best cutoff value of single index was used as the standard, and the patients’ single index greater than or equal to the cutoff value was recorded as a score of 1, otherwise it was a score of 0. The total score of each patient was calculated. Then ROC analysis with the total score of patients (**Figure 5B**) found that the best cutoff value of MSLN(+)CTCs combined with CA125 was 0.5 points, corresponding to an AUC of 0.925, p < 0.0001, with a sensitivity of 92.86% and a specificity of 94.74%. The best cut-off value for MSLN(+)CTCs in combination with HE4 was 0.5 points, corresponding to an AUC of 0.931, p < 0.0001, a sensitivity of 92.31% and a specificity of 84.21%. The best cutoff value for CTCs combined with CA125 was 0.5 points, corresponding to an AUC of 0.940, p < 0.0001, with 100% sensitivity and 84.21% specificity. The best cutoff value for CTCs combined with HE4 was 1.5 points, corresponding to an AUC of 0.970, p < 0.0001, with 76.92% sensitivity and 100% specificity. Finally, we performed a combined diagnosis of the three indicators (**Figure 5C**), when MSLN(+)CTCs were combined with CA125 and HE4, the optimal cutoff value was 1.5 points, corresponding to an AUC of 0.937, p < 0.0001, while the optimal cutoff value of CTCs was 1.5 points when combined with CA125 and HE4, corresponding to an AUC of 0.970, p < 0.0001. Interestingly, the sensitivity and specificity of the two combined methods were the same, 84.62% and 94.74%, respectively. Of all the diagnostic models, only MSLN(+)CTCs in combination with CA125 had a both sensitivity and specificity of more than 90%. The above results indicated that when MSLN(+)CTCs and CTCs were combined as auxiliary diagnostic indicators with CA125 and HE4, the diagnostic efficacy performed better compared to single indicators.

## 4 Discussion

The absence of effective early screening modalities in clinical practice results in approximately 70% of ovarian cancer patients being diagnosed at advanced stages, with a consequent 5-year survival rate of only 20%. However, if early diagnosis is possible, the 5-year survival rate of stage I and II OC can reach 89% and 71% respectively (4). Liquid biopsy is mainly used to detect and analyze circulating tumor cells with the advantages of simplicity, rapidity, reproducibility, minimally invasiveness, and real-time monitoring (37). The CellSearch^®^ system, currently the only Food and Drug Administration (FDA)-approved platform for circulating tumor cells isolation, employs EpCAM-based immunomagnetic enrichment technology (38, 39), and CTCs provide a new way of thinking about diagnosing, treating, and following up on ovarian cancer. However, the diagnostic efficacy of CTCs for ovarian cancer varies due to patient heterogeneity and the diversity of assays (6, 27, 40). Considerable work is still needed for CTCs to become a reliable clinical diagnostic biomarker.

This study represents the investigation incorporating mesothelin, an ovarian cancer-specific target, into circulating tumor cell identification for differentiating benign and malignant ovarian masses. Our results demonstrate promising diagnostic performance, with MSLN(+)CTCs exhibiting superior specificity and conventional CTCs showing higher sensitivity compared to conventional biomarkers (CA125 and HE4) when used individually. In view of the inability of single indicators to achieve satisfactory diagnostic efficacy, we innovatively proposed a simple combined scoring diagnostic model by combining MSLN(+)CTCs and CTCs as auxiliary diagnostic indicators with CA125, HE4 for the diagnosis of ovarian cancer, which ultimately showed superior diagnostic efficacy than that of single indicators. This combinatorial approach demonstrated significantly improved diagnostic efficacy compared to individual indicators. Notably, the combination of MSLN(+)CTCs with CA125 achieved exceptional performance, with both sensitivity and specificity exceeding 90%. This dual-indicators strategy maintained the high specificity characteristic of MSLN(+)CTCs while dramatically improving diagnostic sensitivity.

While our current study focused on differentiating benign and malignant ovarian masses, the findings hold significant promise for early-stage EOC detection. The high specificity of MSLN(+)CTCs (95%) is particularly noteworthy, as current screening modalities like CA125 and transvaginal ultrasound suffer from poor specificity in premenopausal women (12). Notably, our method detected MSLN(+)CTCs in 60% (4/6) of FIGO stage I-II patients (**Supplementary Table S3**), suggesting potential utility in early disease. Future studies should evaluate MSLN(+)CTCs in high-risk asymptomatic populations, particularly BRCA carriers where early detection could significantly impact survival (3, 4). Technical refinements to improve capture efficiency of rare CTC populations and longitudinal monitoring protocols will be essential to realize this potential.

MSLN is a GPI-anchored protein bound to the cell surface that, because of its specific expression in ovarian cancer, also serves as a potential target for antigen-specific therapies as well as chimeric antigen receptor T-cell (CAR-T) therapies (41). Preclinical studies of MSLN-directed CAR-T cells have demonstrated potent anti-tumor activity in ovarian cancer models (42, 43). However, clinical translation has faced several challenges, limiting most trials to phase I/II development. These challenges include genetic heterogeneity within the tumor, poor CAR-T cell migration, antigen escape, insufficient infiltration into the tumor site, and a hostile, highly immunosuppressive tumor microenvironment. As a result, MSLN-directed CAR-T therapies have not yet received FDA approval (41, 44, 45), and significant work remains to overcome these barriers. Meanwhile, in the future, whether MSLN(+)CTCs can be the target of or provide assistance for CAR-T cell therapy targeting MSLN needs to be confirmed by a large amount of work. Overall, MSLN(+)CTCis promising biomarker for both the diagnosis and treatment of ovarian cancer.

There are several shortcomings that need to be addressed in this study. First, the sample size of the enrolled patients was small, limiting subgroup analysis and comparison. We analyzed the difference in the number of MSLN(+)CTCs and CTCs between patients with early and late EOC and did not find a significant difference. This may be a result of the small sample size, or it may be that a sufficient number of CTCs do exist in early stage patients, which would then hold promise for early screening for EOC, and further expansion of the sample size for multicenter validation is needed. Second, the presence of MSLN(+)CTCs or CTCs detection in a few patients with benign lesions may be related to the specificity limitation of the detection method, or it may be that tumor cells do exist in the patient’s body, and such patients should be followed up closely for confirmation. Third, Intermediate populations were not explored in this study, and it is possible that intermediate populations which exist such as CKmix-/DAPI+/CD45-/MSLN+ may be present in the blood of patients. What kind of cell this is and what it does biologically is not yet known, this is an interesting direction for research. Meanwhile, CTCs isolation and detection methods should be further optimized to enhance their sensitivity and specificity. Finally, this article did not address the follow-up of patients’ subsequent treatment efficacy and prognosis. The role of MSLN(+)CTCs in the treatment and prognosis of ovarian cancer patients should be further investigated in the future.

In conclusion, MSLN(+)CTCs represent a highly specific auxiliary biomarker for differentiating benign and malignant ovarian lesions. The combination of MSLN(+)CTCs with CA125 provides an optimal balance between sensitivity and specificity, offering promising clinical utility for EOC diagnosis. In the future, multi-center validation with large samples is needed to further confirm the results of this study.

## Data Availability

The raw data supporting the conclusions of this article will be made available by the authors, without undue reservation.

## References

[1] Webb PM Jordan SJ. Global epidemiology of epithelial ovarian cancer. Nat Rev Clin Oncol. (2024) 21:389–400. doi: 10.1038/s41571-024-00881-3, PMID: 38548868

[2] Sung H Ferlay J Siegel RL Laversanne M Soerjomataram I Jemal A. Global cancer statistics 2020: GLOBOCAN estimates of incidence and mortality worldwide for 36 cancers in 185 countries. CA Cancer J Clin. (2021) 71:209–49. doi: 10.3322/caac.21660, PMID: 33538338

[3] Torre LA Trabert B DeSantis CE Miller KD Samimi G Runowicz CD. Ovarian cancer statistics, 2018. CA Cancer J Clin. (2018) 68:284–96. doi: 10.3322/caac.21456, PMID: 29809280 PMC6621554

[4] Chang L Ni J Zhu Y Pang B Graham P Zhang H. Liquid biopsy in ovarian cancer: recent advances in circulating extracellular vesicle detection for early diagnosis and monitoring progression. Theranostics. (2019) 9:4130–40. doi: 10.7150/thno.34692, PMID: 31281536 PMC6592165

[5] Hacker NF Rao A. Surgery for advanced epithelial ovarian cancer. Best Pract Res Clin Obstetrics Gynaecology. (2017) 41:71–87. doi: 10.1016/j.bpobgyn.2016.10.007, PMID: 27884789

[6] Kim NK Suh DH Kim K No JH Kim YB Kim M. High-throughput viable circulating tumor cell isolation using tapered-slit membrane filter-based chipsets in the differential diagnosis of ovarian tumors. PloS One. (2024) 19:e0304704. doi: 10.1371/journal.pone.0304704, PMID: 38833451 PMC11149860

[7] Zhang R Siu MKY Ngan HYS Chan KKL. Molecular biomarkers for the early detection of ovarian cancer. Int J Mol Sci. (2022) 23:12041. doi: 10.3390/ijms231912041, PMID: 36233339 PMC9569881

[8] Elias KM Guo J Bast RC. Early detection of ovarian cancer. Hematol Oncol Clin North Am. (2018) 32:903–14. doi: 10.1016/j.hoc.2018.07.003, PMID: 30390764 PMC6376972

[9] Huhtinen K Suvitie P Hiissa J Junnila J Huvila J Kujari H. Serum HE4 concentration differentiates Malignant ovarian tumours from ovarian endometriotic cysts. Br J Cancer. (2009) 100:1315–9. doi: 10.1038/sj.bjc.6605011, PMID: 19337252 PMC2676558

[10] Gu Z He Y Zhang Y Chen M Song K Huang Y. Postprandial increase in serum CA125 as a surrogate biomarker for early diagnosis of ovarian cancer. J Transl Med. (2018) 16:114. doi: 10.1186/s12967-018-1489-4, PMID: 29716620 PMC5930842

[11] Szekanecz É Sándor Z Antal-Szalmás P Soós L Lakos G Besenyei T. Increased production of the soluble tumor-associated antigens CA19-9, CA125, and CA15–3 in rheumatoid arthritis. Ann New York Acad Sci. (2007) 1108:359–71. doi: 10.1196/annals.1422.037, PMID: 17893999

[12] Dochez V Caillon H Vaucel E Dimet J Winer N Ducarme G. Biomarkers and algorithms for diagnosis of ovarian cancer: CA125, HE4, RMI and ROMA, a review. J Ovarian Res. (2019) 12:28. doi: 10.1186/s13048-019-0503-7, PMID: 30917847 PMC6436208

[13] Masuda T Hayashi N Iguchi T Ito S Eguchi H Mimori K. Clinical and biological significance of circulating tumor cells in cancer. Mol Oncol. (2016) 10:408–17. doi: 10.1016/j.molonc.2016.01.010, PMID: 26899533 PMC5528976

[14] Lemma S Perrone AM De Iaco P Gasparre G Kurelac I. Current methodologies to detect circulating tumor cells: a focus on ovarian cancer. Am J Cancer Res. (2021) 11(9):4111–26. doi: 10.5281/ZENODO.5800014, PMID: 34659879 PMC8493391

[15] Gallo M De Luca A Maiello MR D’Alessio A Esposito C Chicchinelli N. Clinical utility of circulating tumor cells in patients with non-small-cell lung cancer. Transl Lung Cancer Res. (2017) 6:486–98. doi: 10.21037/tlcr.2017.05.07, PMID: 28904891 PMC5583074

[16] Ciccioli M Kim K Khazan N Khoury JD Cooke MJ Miller MC. Identification of circulating tumor cells captured by the FDA-cleared Parsortix^®^ PC1 system from the peripheral blood of metastatic breast cancer patients using immunofluorescence and cytopathological evaluations. J Exp Clin Cancer Res. (2024) 43:240. doi: 10.1186/s13046-024-03149-x, PMID: 39169412 PMC11337573

[17] Magbanua MJM Carey LA DeLuca A Hwang J Scott JH Rimawi MF. Circulating tumor cell analysis in metastatic triple-negative breast cancers. Clin Cancer Res. (2015) 21:1098–105. doi: 10.1158/1078-0432.CCR-14-1948, PMID: 25524311

[18] Janni WJ Rack B Terstappen LWMM Pierga J-Y Taran F-A Fehm T. Pooled analysis of the prognostic relevance of circulating tumor cells in primary breast cancer. Clin Cancer Res. (2016) 22:2583–93. doi: 10.1158/1078-0432.CCR-15-1603, PMID: 26733614

[19] Zhou J Ma X Bi F Liu M. Clinical significance of circulating tumor cells in gastric cancer patients. Oncotarget. (2017) 8:25713–20. doi: 10.18632/oncotarget.14879, PMID: 28147337 PMC5421964

[20] Yeo D Bastian A Strauss H Saxena P Grimison P Rasko JEJ. Exploring the clinical utility of pancreatic cancer circulating tumor cells. Int J Mol Sci. (2022) 23:1671. doi: 10.3390/ijms23031671, PMID: 35163592 PMC8836025

[21] Goldkorn A Tangen C Plets M Morrison GJ Cunha A Xu T. Baseline circulating tumor cell count as a prognostic marker of PSA response and disease progression in metastatic castrate-sensitive prostate cancer (SWOG S1216). Clin Cancer Res. (2021) 27:1967–73. doi: 10.1158/1078-0432.CCR-20-3587, PMID: 33500355 PMC8026618

[22] Groen L Kloots I Englert D Seto K Estafanos L Smith P. Transcriptome profiling of circulating tumor cells to predict clinical outcomes in metastatic castration-resistant prostate cancer. Int J Mol Sci. (2023) 24:9002. doi: 10.3390/ijms24109002, PMID: 37240349 PMC10218842

[23] Mostert B Sieuwerts AM Bolt-de Vries J Kraan J Lalmahomed Z van Galen A. mRNA expression profiles in circulating tumor cells of metastatic colorectal cancer patients. Mol Oncol. (2015) 9:920–32. doi: 10.1016/j.molonc.2015.01.001, PMID: 25655581 PMC5528769

[24] Vasantharajan SS Barnett E Gray ES McCall JL Rodger EJ Eccles MR. Assessment of a size-based method for enriching circulating tumour cells in colorectal cancer. Cancers. (2022) 14:3446. doi: 10.3390/cancers14143446, PMID: 35884509 PMC9319975

[25] Bhardwaj BK Thankachan S Venkatesh T Suresh PS. Liquid biopsy in ovarian cancer. Clin Chim Acta. (2020) 510:28–34. doi: 10.1016/j.cca.2020.06.047, PMID: 32622965

[26] Kim M Suh DH Choi JY Bu J Kang Y-T Kim K. Post-debulking circulating tumor cell as a poor prognostic marker in advanced stage ovarian cancer. Med (Baltimore). (2019) 98:e15354. doi: 10.1097/MD.0000000000015354, PMID: 31096435 PMC6531062

[27] Guo Y-X Neoh KH Chang X-H Sun Y Cheng H-Y Ye X. Diagnostic value of HE4+ circulating tumor cells in patients with suspicious ovarian cancer. Oncotarget. (2018) 9:7522–33. doi: 10.18632/oncotarget.23943, PMID: 29484129 PMC5800921

[28] Zhang X Li H Yu X Li S Lei Z Li C. Analysis of circulating tumor cells in ovarian cancer and their clinical value as a biomarker. Cell Physiol Biochem. (2018) 48:1983–94. doi: 10.1159/000492521, PMID: 30092594

[29] Blassl C Kuhlmann JD Webers A Wimberger P Fehm T Neubauer H. Gene expression profiling of single circulating tumor cells in ovarian cancer - Establishment of a multi-marker gene panel. Mol Oncol. (2016) 10:1030–42. doi: 10.1016/j.molonc.2016.04.002, PMID: 27157930 PMC5423187

[30] Obermayr E Bednarz-Knoll N Orsetti B Weier H-U Lambrechts S Castillo-Tong DC. Circulating tumor cells: potential markers of minimal residual disease in ovarian cancer? a study of the OVCAD consortium. Oncotarget. (2017) 8:106415–28. doi: 10.18632/oncotarget.22468, PMID: 29290959 PMC5739744

[31] Shen J Sun X Zhou J. Insights into the role of mesothelin as a diagnostic and therapeutic target in ovarian carcinoma. Front Oncol. (2020) 10:1263. doi: 10.3389/fonc.2020.01263, PMID: 32983962 PMC7485315

[32] Wickstroem K Hagemann UB Cruciani V Wengner AM Kristian A Ellingsen C. Synergistic effect of a mesothelin-targeted 227Th conjugate in combination with DNA damage response inhibitors in ovarian cancer xenograft models. J Nucl Med. (2019) 60:1293–300. doi: 10.2967/jnumed.118.223701, PMID: 30850485 PMC6735281

[33] Coelho R Ricardo S Amaral AL Huang Y-L Nunes M Neves JP. Regulation of invasion and peritoneal dissemination of ovarian cancer by mesothelin manipulation. Oncogenesis. (2020) 9:61. doi: 10.1038/s41389-020-00246-2, PMID: 32612258 PMC7329842

[34] Li Y Tian W Zhang H Zhang Z Zhao Q Chang L. MSLN correlates with immune infiltration and chemoresistance as a prognostic biomarker in ovarian cancer. Front Oncol. (2022) 12:830570. doi: 10.3389/fonc.2022.830570, PMID: 35692779 PMC9174524

[35] Hilliard TS Kowalski B Iwamoto K Agadi EA Liu Y Yang J. Host mesothelin expression increases ovarian cancer metastasis in the peritoneal microenvironment. Int J Mol Sci. (2021) 22:12443. doi: 10.3390/ijms222212443, PMID: 34830322 PMC8623331

[36] Ma J Chen Y Ren J Zhou T Wang Z Li C. Purification of circulating tumor cells based on multiantibody-modified magnetic nanoparticles and molecular analysis toward epithelial ovarian cancer detection. ACS Sens. (2023) 8:3744–53. doi: 10.1021/acssensors.3c01063, PMID: 37773014

[37] Zheng X Li X Wang X. Extracellular vesicle-based liquid biopsy holds great promise for the management of ovarian cancer. Biochim Biophys Acta Rev Cancer. (2020) 1874:188395. doi: 10.1016/j.bbcan.2020.188395, PMID: 32698041

[38] Poveda A Kaye SB McCormack R Wang S Parekh T Ricci D. Circulating tumor cells predict progression free survival and overall survival in patients with relapsed/recurrent advanced ovarian cancer. Gynecol Oncol. (2011) 122:567–72. doi: 10.1016/j.ygyno.2011.05.028, PMID: 21664658

[39] Behbakht K Sill MW Darcy KM Rubin SC Mannel RS Waggoner S. Phase II trial of the mTOR inhibitor, temsirolimus and evaluation of circulating tumor cells and tumor biomarkers in persistent and recurrent epithelial ovarian and primary peritoneal Malignancies: a Gynecologic Oncology Group study. Gynecol Oncol. (2011) 123:19–26. doi: 10.1016/j.ygyno.2011.06.022, PMID: 21752435 PMC3336961

[40] Kuo Y-C Chuang C-H Kuo H-C Lin C-T Chao A Huang H-J. Circulating tumor cells help differentiate benign ovarian lesions from cancer before surgery: A literature review and proof of concept study using flow cytometry with fluorescence imaging. Oncol Lett. (2024) 27:234. doi: 10.3892/ol.2024.14367, PMID: 38596263 PMC11003220

[41] Chen Q Sun Y Li H. Application of CAR-T cell therapy targeting mesothelin in solid tumor treatment. Discov Onc. (2024) 15:289. doi: 10.1007/s12672-024-01159-x, PMID: 39023820 PMC11258118

[42] Schoutrop E El-Serafi I Poiret T Zhao Y Gultekin O He R. Mesothelin-specific CAR T cells target ovarian cancer. Cancer Res. (2021) 81:3022–35. doi: 10.1158/0008-5472.CAN-20-2701, PMID: 33795251

[43] Schoutrop E Poiret T El-Serafi I Zhao Y He R Moter A. Tuned activation of MSLN-CAR T cells induces superior antitumor responses in ovarian cancer models. J Immunother Cancer. (2023) 11:e005691. doi: 10.1136/jitc-2022-005691, PMID: 36746513 PMC9906404

[44] Klampatsa A Dimou V Albelda SM. Mesothelin-targeted CAR-T cell therapy for solid tumors. Expert Opin Biol Ther. (2021) 21:473–86. doi: 10.1080/14712598.2021.1843628, PMID: 33176519

[45] Lv J Li P. Mesothelin as a biomarker for targeted therapy. biomark Res. (2019) 7:18. doi: 10.1186/s40364-019-0169-8, PMID: 31463062 PMC6708176

