# Supplementary material for: Insights into the role of MSLN-positive circulating tumor cell as an auxiliary diagnostic biomarker in epithelial ovarian cancer
Source: Front Oncol. 2025 Jul 28;15:1563095. doi: 10.3389/fonc.2025.1563095 (PMC12336444; doi:10.3389/fonc.2025.1563095)

Fig. S1. Expression profiles of manually screened candidate genes in multiple cancer types (red bars) and their corresponding normal tissues (black bars), x-axis shows tissue abbreviations, where “OV” stands for ovarian cancer. Other abbreviations represent different cancer types, y-axis shows gene expression in TPM (transcripts per million).

Tumor vs. Normal:

Tumor vs. Normal

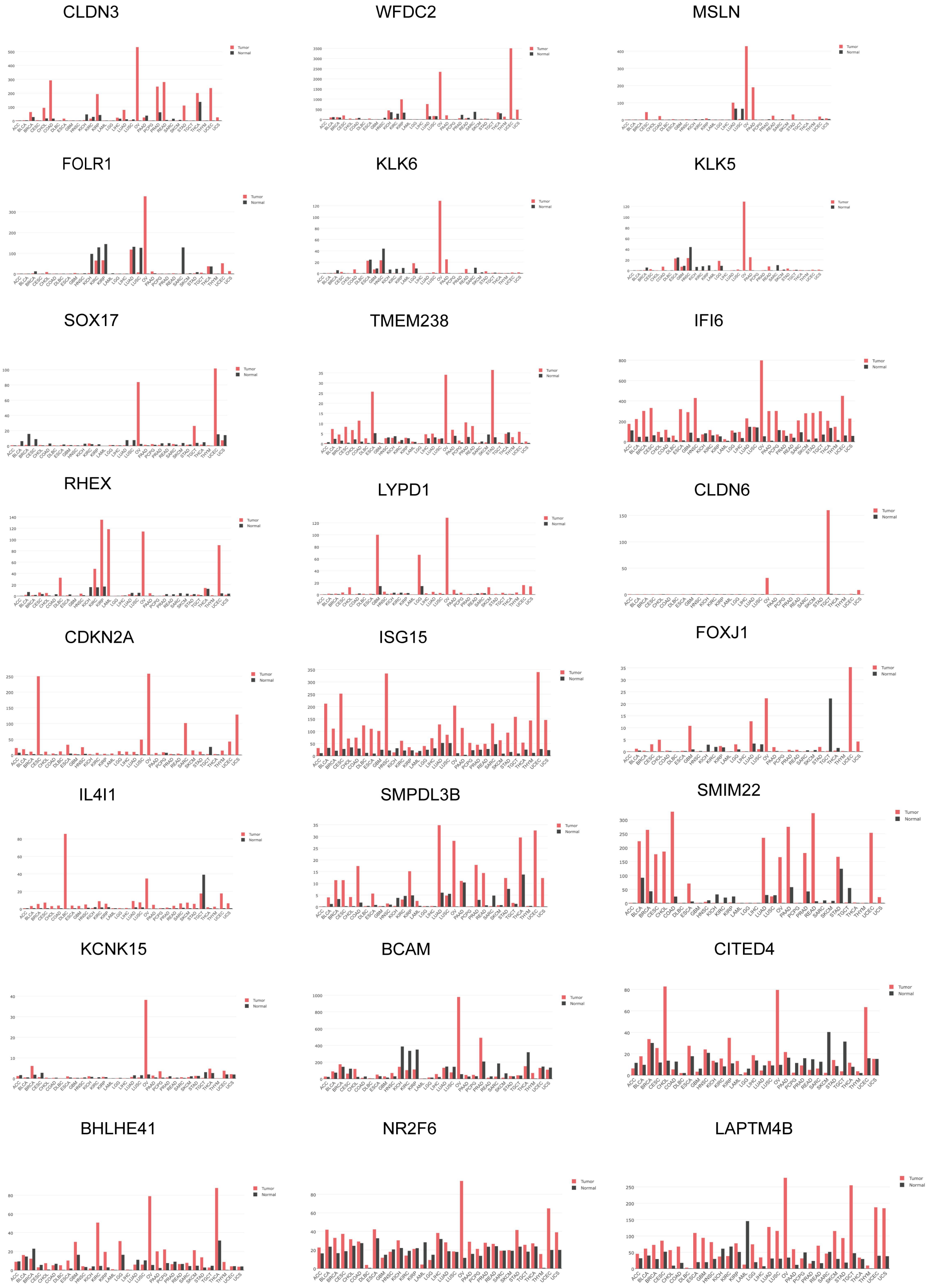

Tumor vs. Normal

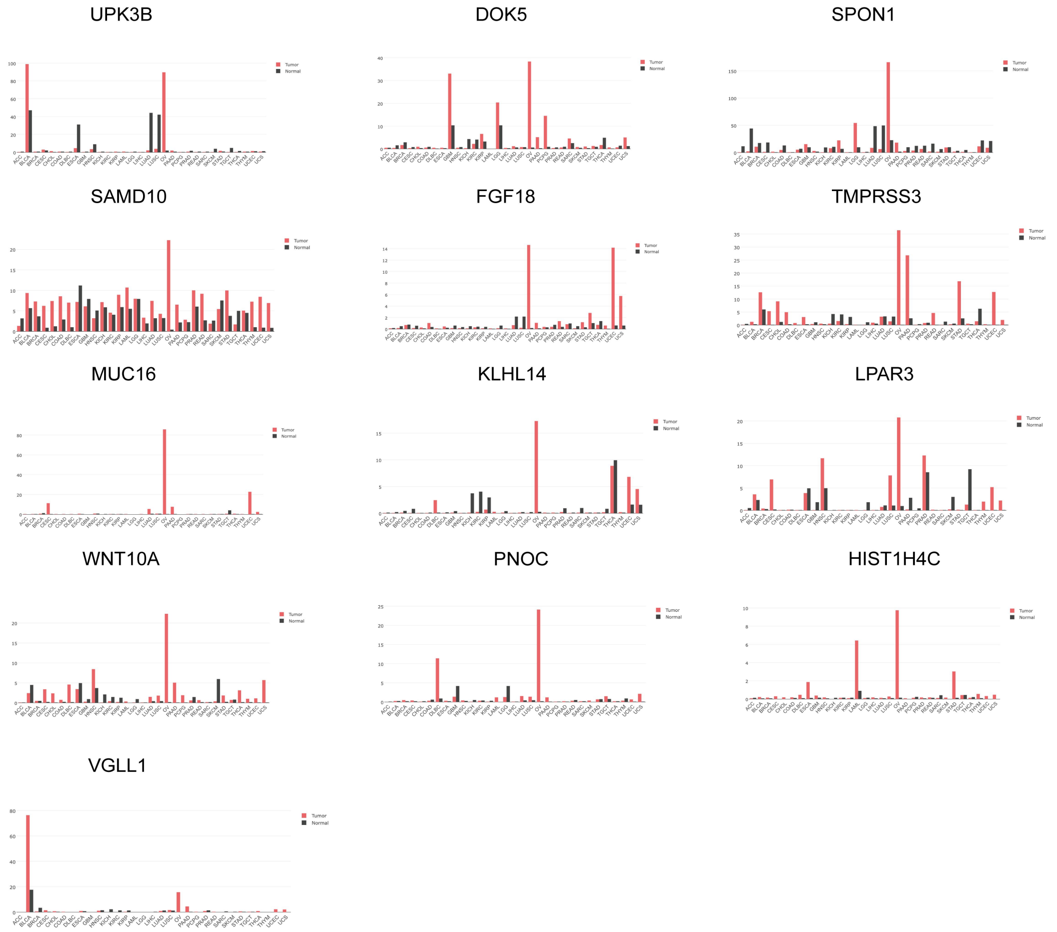

Distant metastasis vs. No metastasis:

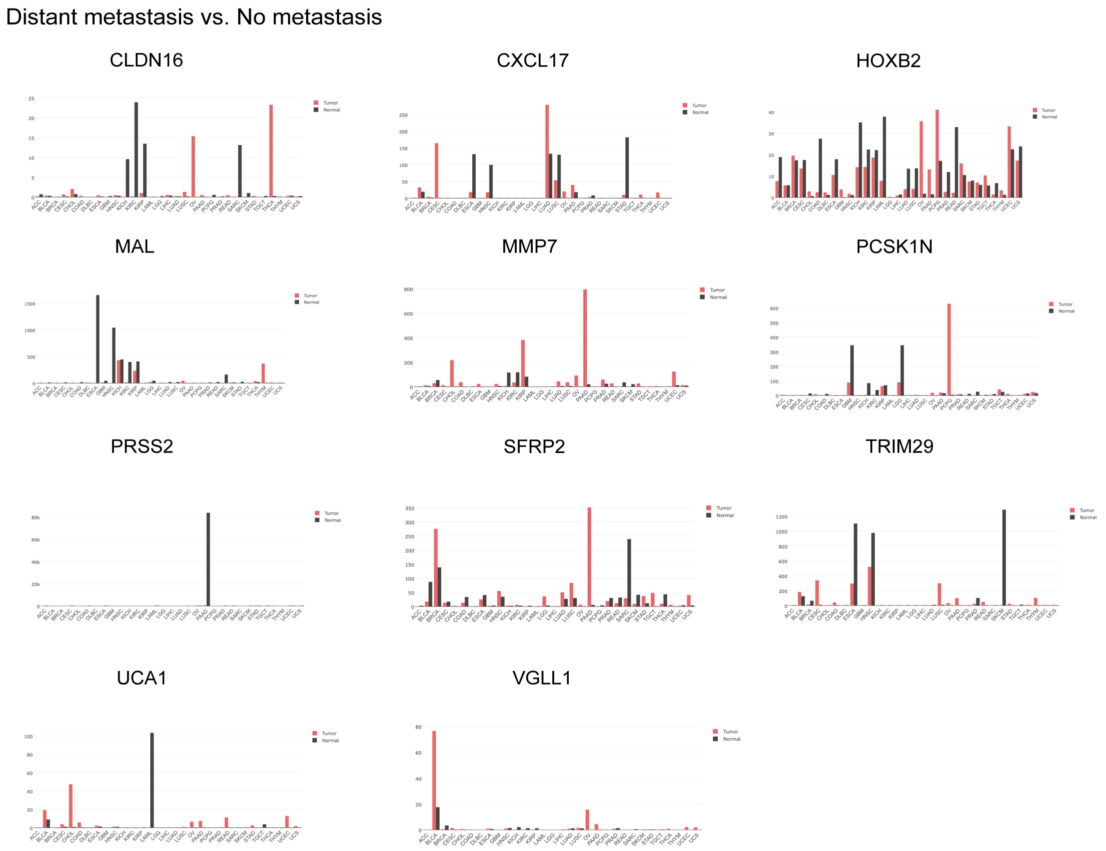

Single-cell sequencing:

Single-cell sequencing selection results

BCAM

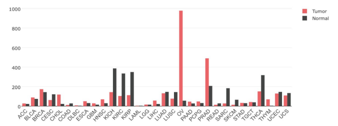

C1ORF186

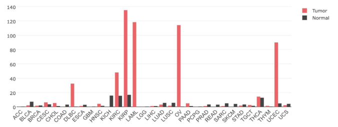

CLDN3

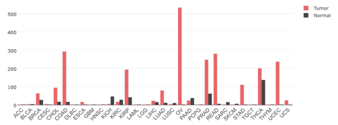

HIST1H4C

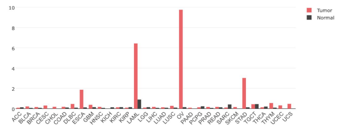

IFI27

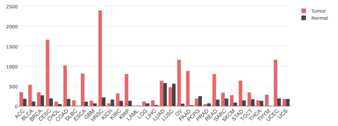

KRT8

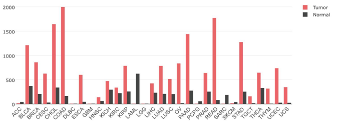

KRT18

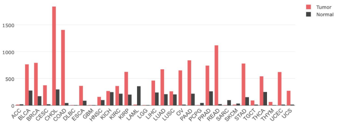

KRT19

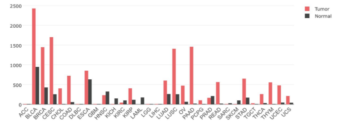

MDK

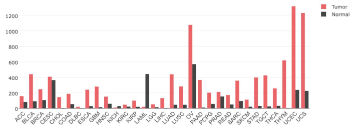

MIF

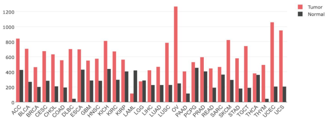

MSLN

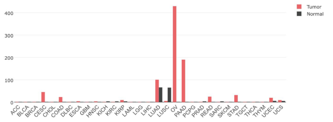

OCIAD2

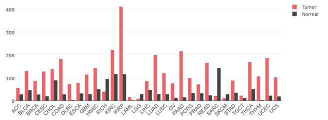

S100A1

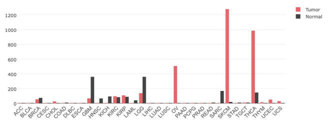

WFDC2

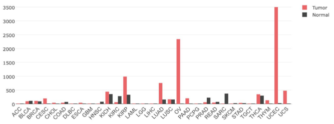

Supplement: Supplementary file 1 [file DataSheet1.pdf]
