# Supplementary material for: Insights into the role of MSLN-positive circulating tumor cell as an auxiliary diagnostic biomarker in epithelial ovarian cancer
Source: Front Oncol. 2025 Jul 28;15:1563095. doi: 10.3389/fonc.2025.1563095 (PMC12336444; doi:10.3389/fonc.2025.1563095)

Table S3. Clinical characteristics of enrolled patients with epithelial ovarian cancer (n=15).

Variable	Value(%)
Age, years	56(37-67)
Initial symptoms	
yes	12(80.0)
no	3(20.0)
CA-125 elevation (≥35 U/mL)	
yes	14(93.3)
no	1(6.7)
FIGO staging	
I-II	6(40.0)
III-IV	9(60.0)
Histology	
Serous carcinoma	9(60.0)
Clear cell carcinoma	5(33.3)
Carcinosarcoma	1(6.7)
lymph node metastasis	
yes	4(26.7)
no	11(73.3)
Peritoneal Metastasis	
yes	7(46.7)
no	8(53.3)

Fig. S2. Patient enrollment. CTC, circulating tumor cell.

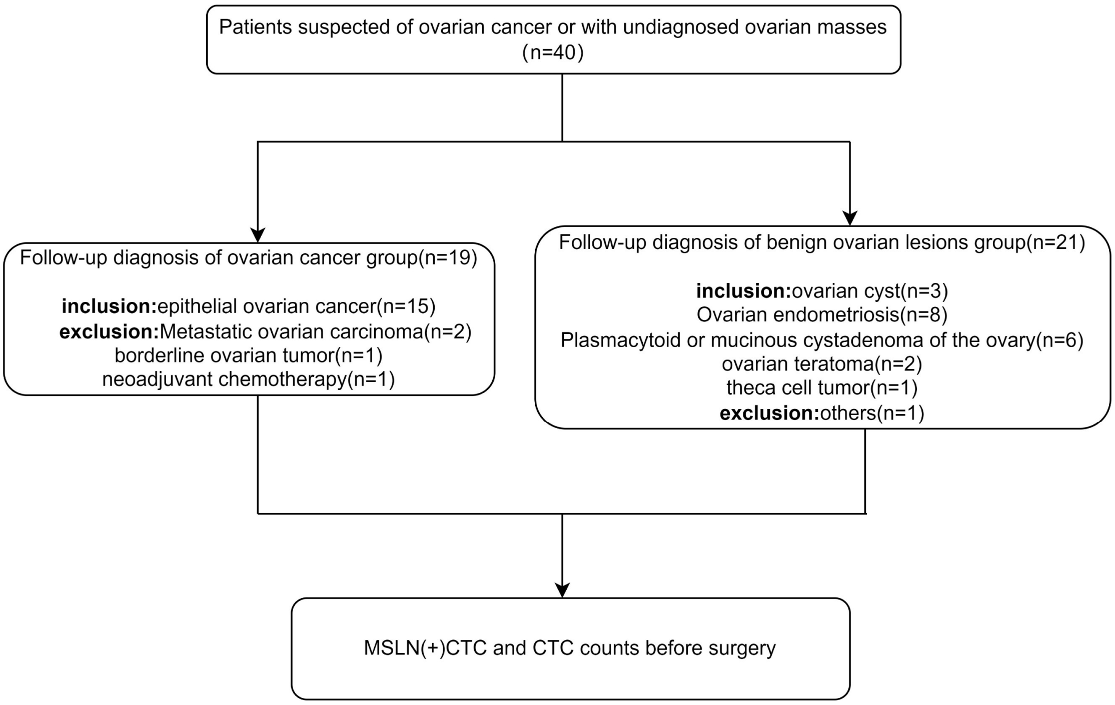

Fig. S3. Scatter plot of age levels of patients with EOC and benign ovarian lesions, the x-axis

is the group and the y-axis is the age range. The age distribution of patients with benign ovarian lesions is shown in gray, and the age distribution of patients with EOC is shown in red. ns, p > 0.05.

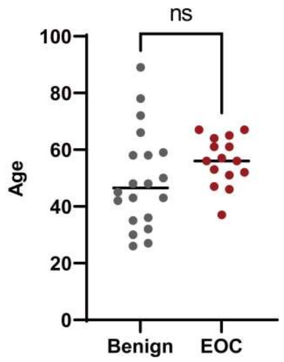

Fig. S4. Scatter plots of MSLN(+)CTC, CTC count and CA125, HE4 levels in patients with EOC and benign ovarian lesions. (A) Scatter plot of MSLN(+)CTC counts (Cells/2mL blood) in patients with EOC and benign ovarian lesions. The x-axis is the group and the y-axis is the range of MSLN(+)CTC number. Distribution of the number of MSLN(+)CTCs in patients with benign ovarian lesions is shown in gray, and the number of MSLN(+)CTCs in patients with EOC is shown in red. (B) Scatter plot of CTC counts (Cells/2mL blood) in patients with EOC and benign ovarian lesions. The x-axis is the group and the y-axis is the range of CTC number. Distribution of the number of CTCs in patients with benign ovarian lesions is shown in gray, and the number of CTCs in patients with EOC is shown in red. (C) Scatter plot of CA125 levels (U/mL) in patients with EOC and benign ovarian lesions. The x-axis is the group and the y-axis is the range of CA125 level. The distribution of CA125 level in patients with benign ovarian lesions is shown in gray, and the distribution of CA125 level in patients with EOC is shown in red. (D) Scatter plot of HE4 levels (pmol/L) in patients with EOC and benign ovarian lesions. The x-axis is the group and the y-axis is the range of HE4 level. The distribution of HE4 level in patients with benign ovarian lesions is shown in gray, and the distribution of HE4 level in patients with EOC is shown in red. ****p < 0.0001.

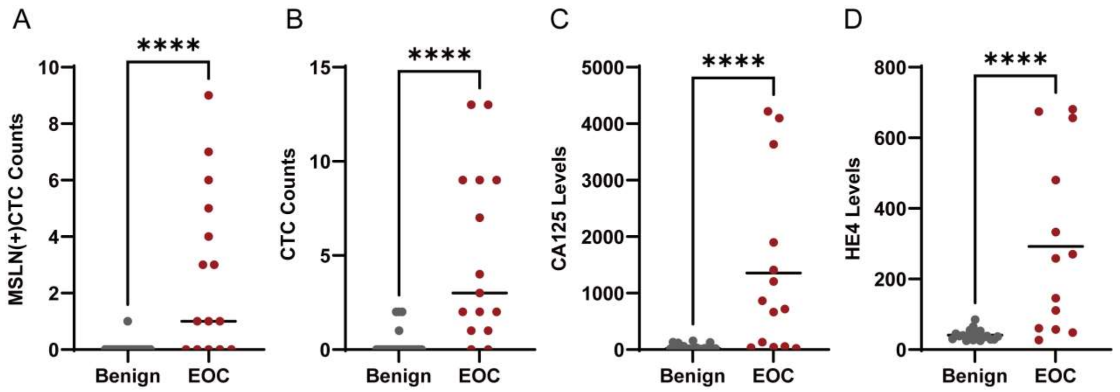

Supplement: Supplementary file 2 [file DataSheet2.pdf]
